# Improvement of antioxidant activity and active ingredient of *Dendrobium officinale via* microbial fermentation

**DOI:** 10.3389/fmicb.2023.1061970

**Published:** 2023-02-08

**Authors:** Gen Yu, QingFen Xie, WenFeng Su, Shuang Dai, XinYi Deng, QuLiang Gu, Shan Liu, JeonYun Yun, WenHao Xiang, Yang Xiong, GuanDong Yang, Yifei Ren, He Li

**Affiliations:** ^1^Key Specialty of Clinical Pharmacy, The First Affiliated Hospital of Guangdong Pharmaceutical University, Guangzhou, China; ^2^Guangdong Provincial Key Laboratory of Pharmaceutical Bioactive Substances, School of Basic Courses, Guangdong Pharmaceutical University, Guangzhou, China; ^3^Guangzhou Base Clean Cosmetics Manufacturer Co., Ltd., Guangzhou, China; ^4^CAS Testing Technical Services (Guangzhou) Co., Ltd., Guangzhou, China; ^5^Guangzhou Huashuo Biotechnology Co., Ltd., Guangzhou, China

**Keywords:** *Dendrobium officinale*, fermentation, condition optimization, antioxidant, GC–MS

## Abstract

This study used brewer’s yeast to ferment *Dendrobium officinale* and single-factor and orthogonal experiments were conducted to determine the optimal fermentation conditions. The antioxidant capacity of *Dendrobium* fermentation solution was also investigated by *in vitro* experiments, which showed that different concentrations of fermentation solution could effectively enhance the total antioxidant capacity of cells. The fermentation liquid was found to contain seven sugar compounds including glucose, galactose, rhamnose, arabinose, and xylose using gas chromatography–mass spectrometry (GC–MS) and high performance liquid chromatography-quadrupole-time of flight mass spectrometry (HPLC-Q-TOF-MS), with the highest concentrations of glucose and galactose at 194.628 and 103.899 μg/ml, respectively. The external fermentation liquid also contained six flavonoids with apigenin glycosides as the main structure and four phenolic acids including gallic acid, protocatechuic acid, catechol, and sessile pentosidine B.

## 1. Introduction

*Dendrobium officinale* is an herb of the genus *Dendrobium* in the family Orchidaceae. It has a long tradition as a traditional Chinese medicine in China and is listed in Shennong’s Materia Medica as a “superior product” (Sun and Sun, 1799). The plant is cylindrical or flattened, about 300 mm long and 4–12 mm in diameter, with a yellow-green, smooth or longitudinally striated surface and conspicuous root nodes, which are covered with membranous leaf sheaths. The inside of the plant is fleshy, sweaty, and easily broken, with a slightly bitter and sweet taste and a sticky texture when chewed. *Dendrobium officinale* is rich in polysaccharides (He et al., 2018), flavonoids (Yuan et al., 2022), alkaloids (Guo et al., 2013), N-containing compounds (Tang, 2015), and many other small-molecule components (Shao et al., 2014). These components make *Dendrobium* have various health effects such as good antioxidant (Zhang et al., 2017), immune system regulation (Xie et al., 2022), fatigue relief (Kim et al., 2020), and reduction of high blood sugar (Fang et al., 2022). Owing to its high nutritional value, *Dendrobium* is often referred to as a life-saving herb among the public.

In recent years, some studies on *Dendrobium officinale* have focused on the extraction (Lei et al., 2021) and bioactivity of functional components (Xiao et al., 2011). Most of them focused on the study of *Dendrobium officinale* extract, and less on the study of *Dendrobium* fermentation. Zhu et al. (2019) determined the optimal extraction conditions of quercetin in *Dendrobium* by central composite design response surface method as ethanol concentration 81.6%, the material-liquid ratio is 1:41.1 g/ml, 30 min fermentation time, and 30 min ultrasonic extraction at power 160 W. Tao et al. (2021) found that polysaccharides in *Dendrobium* had good anti-tumor effects *in vivo* and enhanced the anti-cancer effect of 5-fluorouracil (5-Fu). Chen et al. (2022) studied that flavonoids in *Dendrobium officinale* had the ability to protect cells from ultraviolet (UVB) damage. Meanwhile, Tian et al. (2019) used Bacillus to ferment *Dendrobium officinale*, The Mw and monosaccharide composition of *Dendrobium officinale* polysaccharide was changed and the immunomodulatory activity of Dendrobium officinale polysaccharide was enhanced after the strain fermentation. Li et al. (2021) used brewer’s yeast to ferment apples to enrich the antioxidant capacity and nutritional properties of apple juice. No studies are still used brewer’s yeast to ferment *Dendrobium* and to investigate the structure and efficacy of the functional components of the fermentation liquid. The purpose of this study was to systematically optimize the fermentation process of *Dendrobium officinale*, analyze the antioxidant capacity of the fermentation products, and identify the chemical components contained in the fermentation products. The results of the study will lay the foundation for the scientific development of the *Dendrobium* industry.

## 2. Materials and methods

### 2.1. Materials and reagents

*Dendrobium* was purchased from Guangzhou Fuzilin Biotechnology Co. The Bradford protein quantification kit, total antioxidant capacity (T-AOC) test kit, malondialdehyde (MDA) test kit, total superoxide dismutase (T-SOD) assay kit, and catalase (CAT) assay kit were purchased from Nanjing Jiancheng Institute of Biological Engineering; The brewer’s yeast used in the trials was obtained from our own laboratory; All other reagents were analytically pure.

### 2.2. Optimization of the *Dendrobium officinale* fermentation process

In this study, single-factor experiments were utilized to optimize the fermentation process and antioxidant capacity was used as the evaluation standard of this study. Six factors, namely the optimum carbon source, carbon source concentration, fermentation temperature, fermentation days, bacteria additional amount, and material-to-liquid ratio (Table 1) were selected for single-factor experiments. Inspired by the results of the single-factor experiments, orthogonal experiments were conducted to select the significant influencing factors and carry out response surface optimization to obtain the optimal fermentation conditions for *Dendrobium*. The Fenton reaction method (H_2_O_2_+Fe^2+^=-OH+H_2_O+Fe^3+^) is commonly used to determine the antioxidant capacity (Yeung et al., 2021). The reaction produces pyrocatechic acid through the reaction between salicylic acid and hydroxyl radicals, and the reactants have a maximum absorption wavelength of 510 nm. The hydroxyl radical scavenging ability of the fermentation liquid from different strains was determined by adding reagents to each group as shown in Table 2. After heating in a water bath at 37°C for 15 min, the absorbance was measured at OD510 nm, and the hydroxyl radical scavenging rate (%) = $\left[A_{0}-\left(A_{X}-A_{X0}\right)\right]/A_{0}\times 100\%$.

**Table 1:** Single factor experiment on fermentation of *Dendrobium officinale*.

Factor	Level	Evaluation
Carbon source	Lactose, Maltose, Glycerin, Fructose, Glucose	Hydroxyl radical scavenging rate
Carbon source concentration (%)	2, 4, 6, 8, 10	
Fermentation temperature (°C)	4, 25, 37, 50	
Fermentation days (days)	1, 2, 3, 4, 5	
Solid-liquid ratio	1:4,1:5,1:6,1:7,1:8	
Bacteria addition amount (ml)	2, 4, 5, 6, 8, 10	

**Table 2:** Experimental procedure for hydroxyl radical scavenging rate.

Reagents	Content/ml		
	*A_0_*	*A_x_*	*A_x0_*
9 mmol/LFeSO_4_	1	1	1
9 mmol/LSalicylic acid	1	1	1
Sample	0	2	2
Deionized water	12	10	11
8.8 mmol/LH_2_O_2_	1	1	0

### 2.3. *In vitro* antioxidant activity of *Dendrobium* fermentation liquid

Human skin fibroblasts (HSF cells) were selected for the experiment. 1 ml of resuscitated HSF cell suspension was inoculated into a T25 culture flask, and 4 ml of DMEM medium containing 15% bovine embryo serum was added for culture. The cells were cultured at 37°C and 5% CO_2_ until the cell coverage rate was greater than 80%. 100 μl cell suspension at the logarithmic growth stage was added with 0.4% trypan blue chromogenic solution for chromogenic development. The number of cells on the cell counting plate was counted, and then the cell suspension was diluted to 1 × 10^5^ Cells/ml. And the samples of *Dendrobium* fermentation liquid at 0.5, 1, 2, 4, 6, 8, 10, and 20% (v/v) concentrations were prepared from the medium.

#### 2.3.1. Toxic effects of fermentation liquid on cells

In order to confirm the safety of *Dendrobium officinale* fermentation broth in cells, this study used the Thiazolyl blue tetrazolium bromide colorimetric assay to determine the safe concentration of the fermentation broth (Sun et al., 2019). The edge wells of the 96-well plate were full of sterile phosphate buffered saline (PBS) and the rest of the wells were divided into 6 parallel groups of experimental groups, a blank group, and a control group. 100 μl of cell suspension (1×10^5^ Cells/ml) was added to the experimental groups and control group which incubated at 37°C for 24 h under 5% CO_2_. 100 μl of different concentrations of fermentation liquid [0.5, 1, 2, 4, 6, 8, 10, and 20% (v/v)] was added to the experimental group and only DMEM was added to the control group. The experimental group was incubated with 100 μl of different concentrations of fermentation liquid [0.5, 1, 2, 4, 6, 8, 10, and 20% (v/v)], the control group was only replaced with DMEM, and the blank group was incubated with only DMEM. 4 h before the end of the incubation, 50 μl of MTT(3-(4,5-dimethylthiazol-2-yl)-2-5-diphenytetra zolium bromide) was added to each well and the incubation was continued for 4 h to reduce MTT to methanogen The supernatant was then aspirated and 150 μl of dimethyl sulfoxide (DMSO) was added and shaken at low speed. The absorbance was measured at 570 nm. The effect of different concentrations of *Dendrobium* fermentation liquid on the survival rate of HSF (%) is calculated according to $\text{Cell survival rate}\left(\%\right)=\left(A_{X}-A_{0}\right)/\left(A_{X0}-A_{0}\right)$. In which *A_X_* indicates sample test group; *A_0_* indicates blank group and *A_X0_* indicates control group. The appropriate fermentation liquid concentration was obtained.

#### 2.3.2. The model of oxidative damage in HSF cells

The cell suspension was adjusted to 1 × 10^5^ cells/ml, 100 μl of the cell suspension was added to a 96-well plate, and the marginal wells were filled with PBS, the supernatant was discarded and after incubation at 37°C for 24 h at 5% CO_2_. The model of oxidative damage included the experimental groups, the blank group, and the control group. The experimental group (0.2, 0.4, 0.5, 0.6, 0.7, 0.8, 0.9, and 1.0 mmol/L hydrogen peroxide) was added 100 μl hydrogen peroxide at different concentrations per well, replacing H_2_O_2_ with DMEM only in the control group and adding DMEM only in the blank group. 6 parallel experiments were set up for each concentration, and 50 μl of MTT staining solution was added to each well in turn after 20 h. After 4 h of incubation, the supernatant was discarded, then 150 μl of DMSO was added, and the absorbance value at 750 nm was measured at low speed for 10 min, The survival rate of HSF cells was calculated according to the $\text{Cell survival rate}\;\left(\%\right)=\left(A-A_{0}\right)/\left(A_{1}-A_{0}\right)$. In Which *A* is the absorbance value of the experimental group, *A_0_* is the absorbance value of the blank group and *A_1_* is the absorbance value of the control group.

#### 2.3.3. Vitro antioxidant in *Dendrobium* fermentation solution

Prevention group: divided into blank group, control group, model group, low, medium, and high concentration groups of *Dendrobium* fermentation (low concentration group is 0.5%; medium concentration group is 1%; high concentration group is 2%), adjust the cell suspension concentration to 1 × 10^5^ cells/ml, add 2 ml cell suspension to each group, incubate at 37°C, 5% CO_2_ for 24 h. Discard the supernatant, add 2 ml low, medium, and high concentrations of dendrobium fermentation solution to the experimental group, 2 ml medium to the model group and blank group, and 2 ml positive group Vitamin c (Vc) culture solution was added to the positive group, and after 20 h of action, the supernatant was discarded, and 2 ml of 0.5 mmol/L hydrogen peroxide was added to each well for 4 h, and the supernatant was discarded to digest the cells. After digestion, the cells were centrifuged at 1000 r/min for 10 min, blown out with 1 ml PBS and sonicated at 4°C for 5 min (100 W, 5 s/time, 20s interval). Cellular protein concentration was measured.

Treatment group: After 24 h of cell culture according to the above experimental procedure, The difference with the prevention group is 0.5 mmol/L H_2_O_2_ was added first to construct the injury model, then low, medium, and high concentrations of *Dendrobium* fermentation solution were added, and the rest of the experimental procedure remained unchanged.

##### 2.3.3.1. Determination of total antioxidant capacity (T-AOC) of fermentation liquid *in vitro*

The samples were treated with the Total Antioxidant Capacity (T-AOC) test kit and then reacted at room temperature for 6 min. The OD value of each well was measured at 405 nm and substituted into the standard curve to obtain the total antioxidant capacity of the fermentation liquid.

##### 2.3.3.2. Determination of malondialdehyde (MDA) content

The samples were treated with the malondialdehyde (MDA) kit, mixed after the addition of reagents, and centrifuged at 4000 r/min for 10 min after cooling in a water bath at 95°C for 40 min. The supernatant was extracted and the absorbance was measured at 532 nm to calculate the content according to the formula.

##### 2.3.3.3. Total superoxide dismutase activity determination

Fermentation liquids were treated with Total Superoxide Dismutase (T-SOD) kit, reacted at room temperature for 10 min and absorbance values were measured at 550 nm. SOD activity was calculated according to the formula.

##### 2.3.3.4. Determination of catalase activity

The samples were treated with a catalase (CAT) test kit and the absorbance was measured at 240 nm by UV spectrophotometer and CAT activity was calculated according to Equation.

### 2.4. Determination of the composition of *Dendrobium officinale* fermentation liquid

#### 2.4.1. Polysaccharide in *Dendrobium officinale* fermentation liquid

The fermentation solution was added to distilled water at a ratio of 1:30 and extracted twice at 70°C for 2 h. The extraction solution was collected and concentrated under reduced pressure, anhydrous ethanol was added to make the final concentration to 80%, refrigerated for 12 h to separate and precipitate the crude polysaccharide, sevag reagent (chloroform: n-butanol = 5:1) was added to precipitate the denatured protein in it, and the protein precipitation between the aqueous layer and the organic layer was repeated several times until there was no protein precipitation to obtain *Dendrobium officinale* polysaccharide solution, and GC–MS was used to determine the polysaccharide species and H_2_O), mixed well and left to stand at room temperature for 1 h. The reaction was then suspended by the addition of acetic acid. To the above reaction solution, N-methylimidazole and acetic anhydride were added and the derivatization was carried out at room temperature for 1 h. The reaction was then aborted by the addition of distilled water. 2 ml of trichloromethane was added for liquid–liquid extraction, and the lower organic phase was washed with distilled water and passed through a 0.22 μm filter membrane. 1 μl was injected into the sample at a carrier gas (helium) flow rate of 1 ml/min on an AB-5 column (30 m × 250 μm × 0.25 μm). Inlet 250°C, transfer line 280°C, ion source 230°C, and quadrupole 150°C. The temperature was ramped up (starting at 100°C; heating to 200°C for 1 min at 5°C/min followed by heating to 280°C for 5 min at 10°C/min).

The mass spectrometry was performed with a Dual AJS ESI ion source with positive and negative ion scanning; drying gas (N_2_) at 300°C and a flow rate of 8 L/min; nebulizing gas (N_2_) at 35 psi; sheath gas at 350°C and a flow rate of 11 L/min; electrospray voltage of 3,500 V; capillary exit voltage of 150 V; cone hole voltage of 65 V; octupole voltage of 750 V; scanning Range: m/z 100–1,000; collision energies 10, 20, 40 eV.

#### 2.4.2. Flavonoids in *Dendrobium officinale* fermentation liquid

The fermentation product is added to 80% ethanol at a ratio of 1:30, sonicated at 200 W for 30 min, and filtered. The filtrate was filtered by adding 80% ethanol at a ratio of 1:20 and sonicated at 200 W for 30 min. The filtrate was filtered by adding 80% ethanol at a ratio of 1:10 and sonicating at 200 W for 30 min. The filtrate was combined to obtain the crude extract of flavonoids, which was left at 4°C overnight and filtered. The filtrate was concentrated by a rotary evaporator (−0.1 MPa, 80°C) to obtain the flavonoid extract. The fermentation liquid was analyzed by HPLC-Q-TOF-MS on a Waters HSS-T3 column (150 × 2.1 mm, 3.5 μm) using 0.1% formic acid as mobile phase A and acetonitrile as mobile phase B. The extract was centrifuged at 10,000 r/min for 10 min and the supernatant was filtered through a 0.22 μm membrane. Gradient elution (0–3 min, 0% B; 3–10 min, 0–10% B; 10–20 min, 10–40% B; 20–30 min, 40–80% B; 30–32 min, 80% B; 32.1–35 min, 0% B). After Agilent Dual AJS ESI ion source, positive and negative ion scanning (drying gas (N_2_) temperature 300°C, flow rate 8 L/min; nebulizing gas (N_2_) pressure 35 psi; sheath gas temperature 350°C; sheath gas flow rate 11 l/min; elect rospray voltage 3,500 V; capillary outlet voltage 150 V; cone hole voltage 65 V; octopole voltage 750 V; scan range:m/z 100-1,000; collision energies 10, 20, 40 eV) to obtain results.

#### 2.4.3. Polyphenols in *Dendrobium officinale* fermentation liquid

The fermentation was carried out according to the conditions obtained from the response surface optimization, and the resulting fermentation liquid was added to 120 ml of 70% ethanol and placed in a constant temperature water bath at 60°C and extracted twice at reflux for 1 h. The extracts were collected by filtration and concentrated to obtain the phenol extracts. The analytical method was the same as in 2.4.2.

### 2.5. Data statistics

All samples from the different processes were tested in three replicates. Data were assessed as mean ± standard deviation (SD). Analysis of variance (ANOVA) was performed using Design-Expert 10.0 and the differences were statistically significant (*p* < 0.05).

## 3. Results and discussion

### 3.1. Optimization of fermentation conditions for *Dendrobium officinale*

#### 3.1.1. Results of the single-factor experiment

**Figure 1:**
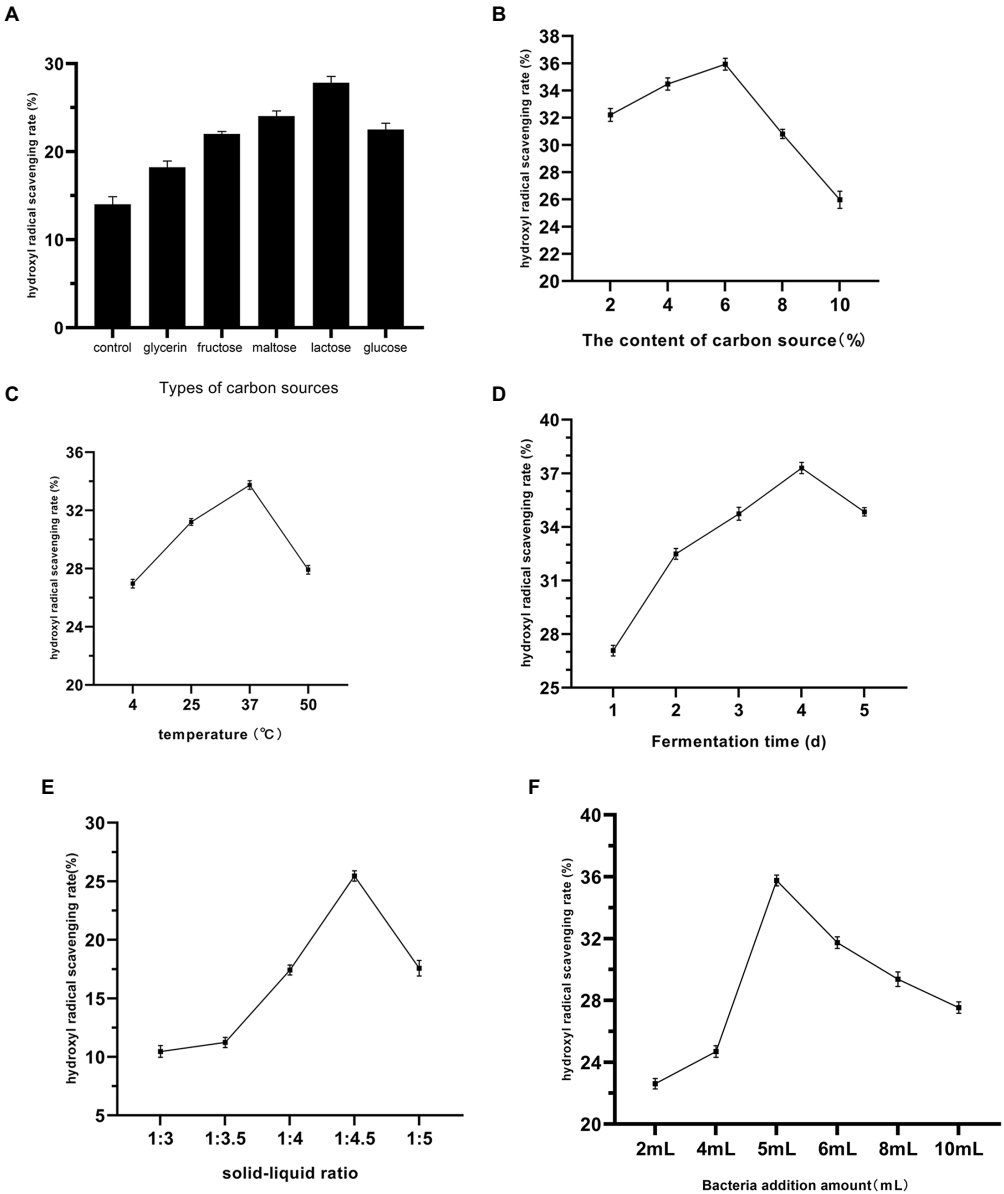
Effects of different carbon source types **(A)**, carbon source concentration **(B)**, fermentation temperature **(C)**, fermentation time **(D)**, material-liquid ratio **(E)**, and Bacteria addition amount **(F)** on the fermentation of *Dendrobium officinale.*

From Figure 1A, the addition of different types of carbon sources to the fermentation system significantly increased the hydroxyl radical scavenging rate of the fermentation liquid, and when the carbon source was lactose, the hydroxyl radical scavenging rate of the fermentation liquid reached a maximum of 27.84%, which was twice as high as that of the blank group. The addition of the carbon source provided the yeast with sufficient carbon to ensure cell growth and development (Martínez-Pastor and Estruch, 1996), which facilitated the metabolism of metabolites with antioxidant capacity. In Figure 1B, the hydroxyl radical scavenging rate of the fermentation liquid gradually increased at 2 to 6% lactose concentration, reaching the highest value at 6% lactose concentration. In Figure 1C, 4, 25, 37, and 50°C were chosen to indicate the influence of low temperature, room temperature, and high temperature on the fermentation of *Dendrobium officinale*, and the hydroxyl radical scavenging rate of the fermentation liquid reached the highest level at 37°C. This indicates that 37°C is the best temperature for the fermentation of *Dendrobium* in antioxidant potential. Figure 1D shows that the hydroxyl radical scavenging rate of the fermentation liquid gradually increased with the increase of fermentation time, reaching the maximum value at 4 days, and the activity of the fermentation liquid decreased due to competitive inhibition between too many strains as the fermentation time increased. In Figure 1E, different stock ratios also had different effects on the fermentation liquid, with low stock ratios being favorable to the increase of hydroxyl radical scavenging rate in the fermentation liquid, and the best effect was achieved at a stock ratio of 1:4.5. The hydroxyl radical scavenging rate of the fermentation liquid was maximized at 5 ml of strains added (Figure 1F), and the hydroxyl radical scavenging rate of the fermentation liquid decreased gradually due to the competition between the strains in excess of 5 ml.

#### 3.1.2. Results of the orthogonal test

**Figure 2:**
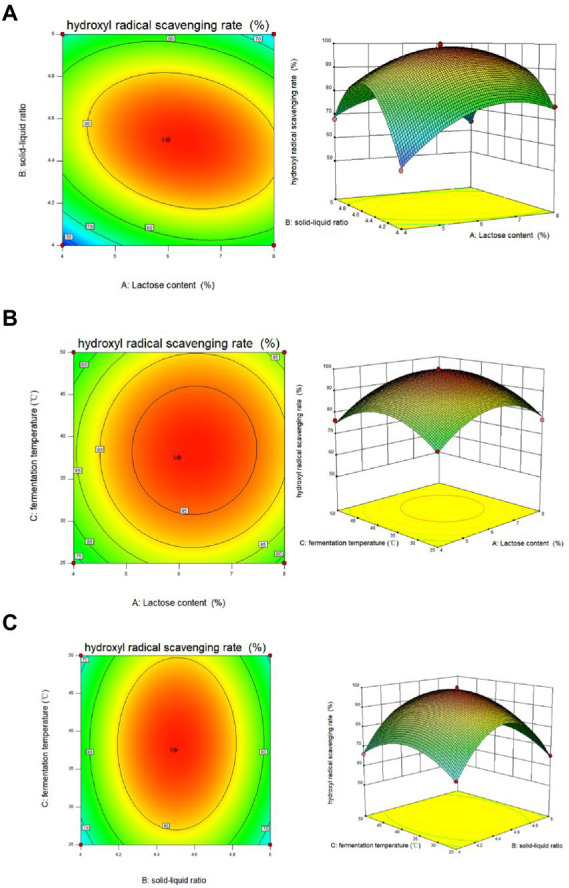
Response surface and contour plots of **(A)** lactose content, **(B)** solid–liquid ratio and **(C)** fermentation temperature on the hydroxyl radical scavenging rate of *Dendrobium officinale* fermentation broth.

From the results of the single-factor test, the factors and levels were determined as follows: Factor A was the feed-to-liquid ratio (1:4 at low level and 1:5 at high level), Factor B was the length of fermentation (3 days at low level and 5 days at high level), Factor C was the amount of strain added (4 ml at low level and 6 ml at high level), Factor D was the fermentation temperature (25°C at low level and 50°C at high level) and Factor E was the lactose content (4% at low levels and 8% at high levels). The Plackett-Burman (P-B) experiment was used to analyze the significant factors affecting the *Dendrobium* fermentation liquid, resulting in a multivariate primary equation for the relationship between the five factors and the relative hydroxyl radical scavenging rate of *Dendrobium* fermentation liquid: R1 = +66.60 + 10.71A + 5.93B + 0.62C + 12.40D + 2.10E, with R1 denoting the relative hydroxyl radical scavenging rate. The analysis of variance (Table 3) showed factor D > factor A > factor E > factor C > factor B, with *p* = 0.0005 for the model, indicating significance at the 95% confidence level, indicating that fermentation temperature (*p* = 0.0002), feed to liquid ratio (*p* = 0.0005), and lactose content (*p* = 0.0088) had a the three factors were chosen to design the Box. The Box-Behnken (B-B) test was designed with these three factors for response surface analysis, and the regression model was obtained as: R1 = +98.61 + 3.16A + 0.55B + 1.42C-6.13AB + 0.48 AC + 0.36 BC-11.09A^2^-22.04B^2^-10.44C^2^, with R1 indicating the relative hydroxyl radical scavenging rate, A, B, and C refer to lactose content, feed to liquid ratio and fermentation temperature, respectively. Response surface analysis showed (Table 4) that *p*-value for the model = 0.0113 < 0.05 (significant); the misfit term *p* = 0.0507 > 0.05 (insignificant) indicated that the modified model fitted the experiment well with little error. The response surface and contour plots (Figure 2) predict that the optimum level of each factor is 8.00% lactose, 37.50°C fermentation temperature, and 1:4 feed-to-liquid ratio, and the peak hydroxyl radical scavenging rate was predicted to be 84.77%. The model was then validated and the hydroxyl radical scavenging rate was 88.30%. The difference between the predicted value and the experimental value was small, which proved that the model was reliable comparing the hydroxyl radical scavenging rate before and after fermentation optimization, we found that the hydroxyl radical scavenging rate after optimization increased 1.28 times compared with that before optimization, from 38.73 to 88.30%.

**Table 3:** ANOVA results.

Factor	Sum of squares	Degrees of freedom	Mean square	*F-value*	*p-value*	Significance ranking
Model	3689.42	5	737.88	26.30	0.0005	*
A – Material to liquid ratio	1333.74	1	1333.74	47.53	0.0005	2
B – Duration of fermentation	4.50	1	4.50	0.16	0.7027	5
C – Amount of strain added	51.16	1	51.16	1.82	0.2256	4
D – Fermentation temperature	1788.06	1	1788.06	63.72	0.0002	1
E – Lactose	408.85	1	408.80	14.57	0.0088	3
Residual	168.36	6	28.06			
Total	3857.78	11				

* Significant at 95% confidence level.

**Table 4:** Response surface ANOVA results.

Factor	Sum of squares	Degrees of freedom	Mean square	*F-value*	*p-value*	Significance ranking
Model	3470.11	9	385.57	180.17	<0.0001	*
A-Lactose	39.74	1	39.74	18.57	0.0035	
B-Material to liquid ratio	17.55	1	17.55	8.20	0.0242	
C-Fermentation temperature	16.13	1	16.13	7.54	0.0287	
AB	255.52	1	255.52	119.40	<0.0001	*
AC	0.92	1	0.92	0.43	0.5326	
BC	0.52	1	0.52	0.24	0.6376	
A^2^	434.53	1	434.53	203.05	<0.0001	*
B^2^	1875.23	1	1875.23	876.28	<0.0001	*
C^2^	544.92	1	544.92	254.64	<0.0001	*
Residual	14.98	7	2.14			
Lack of fit	7.28	3	2.43	1.26	0.3999	
Pure error	7.70	4	1.92			
Cor total	3485.09	16				

* Significant at 95% confidence level.

### 3.2. *In vitro* antioxidant results of *Dendrobium* fermentation liquid

**Figure 3:**
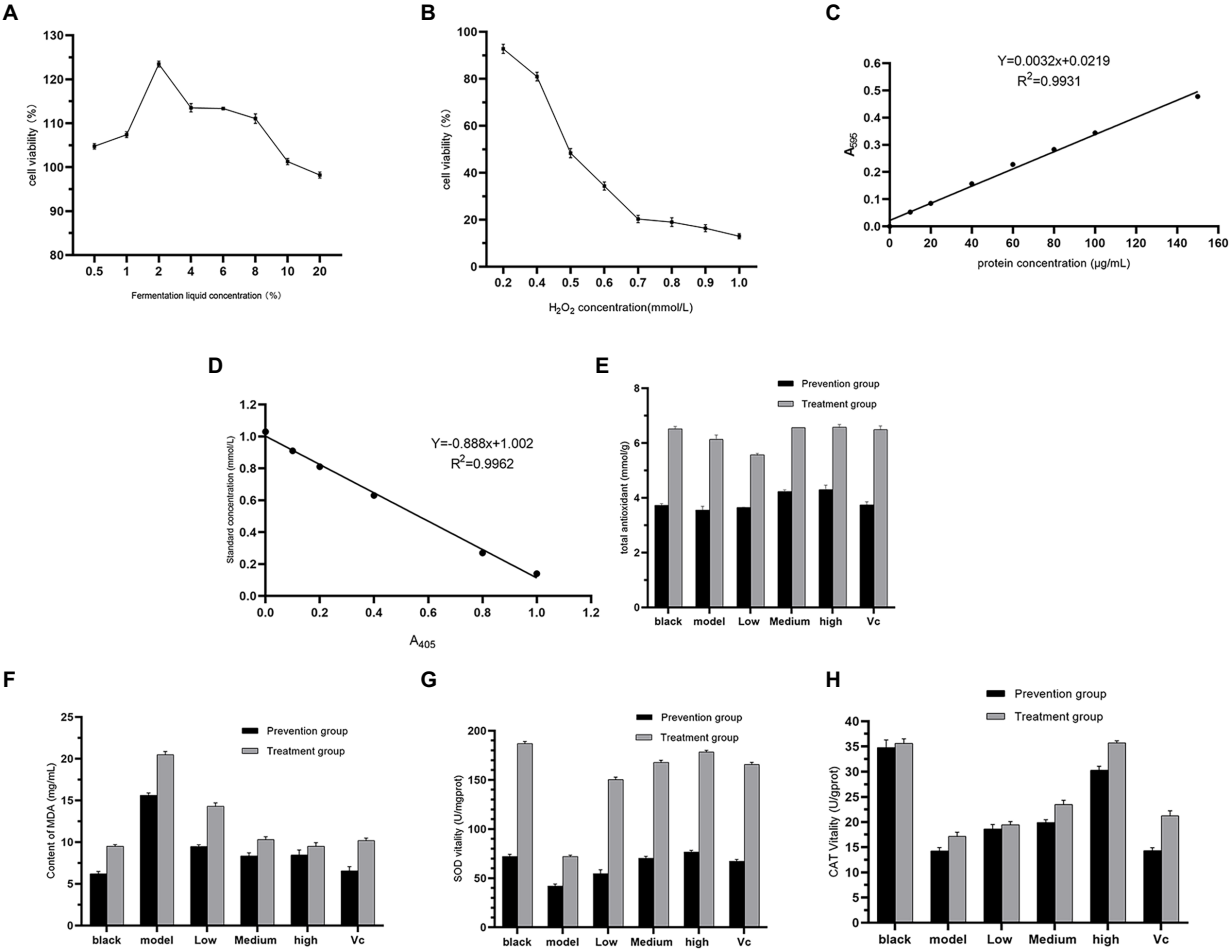
Results of fermentation liquid concentration **(A)**, H_2_O_2_ concentration **(B)**, protein standard curve **(C)**, total antioxidant capacity standard curve **(D)**, total cellular antioxidant capacity **(E)**, MDA content **(F)**, SOD activity **(G)**, CAT activity **(H)** in cell assays.

The Thiazolyl blue tetrazolium bromide colorimetric assay is commonly used as one of the methods to assess cytotoxicity (Jyoti et al., 2021), and from Figure 3A, cell viability gradually increases at fermentation liquid concentrations of 0.5 to 2%. Above 2%, cell survival gradually decreased. Therefore, 0.5, 1, and 2% concentrations were used as the low to medium to high concentration groups. Hydrogen peroxide (H_2_O_2_) acts as a reactive oxygen cluster radical that damages intracellular protein structure (Gentilin et al., 2021) leading to cell damage, and this study used H_2_O_2_ to construct an *in vitro* oxidative damage model. In Figure 3B, H_2_O_2_ concentrations in the range of 0.2 to 1.0 mmol/l inhibited cell growth. At 0.5 mmol/L H_2_O_2_, the cell survival rate was 50.75%. At concentrations greater than 0.5 mmol/L, the cell survival rate was less than 40% and the cell damage was severe, so 0.5 mmol/L H_2_O_2_ was chosen to construct the cell damage model.

From the standard curves of protein concentration (Figure 3C) and total antioxidant capacity (Figure 3D), it can be seen that after the effect of the fermentation solution, the cell T-AOC of the experimental group with medium and high concentration increased compared with the model group, and the effect of high concentration of fermentation solution T-AOC was comparable to that of the control group (V_C_; Figure 3E). This indicates that *Dendrobium officinale* fermentation liquid has a good therapeutic effect on cells damaged by free radicals. The MDA content, a marker of cellular lipid peroxidation (Dharmajaya and Sari, 2022), was used to evaluate the antioxidant capacity of the fermentation liquid, and in Figure 3F, the low, medium and high concentration experimental groups significantly reduced the MDA content (*p* < 0.05), with the effect increasing as the concentration increased. The overall MDA content of the prevention group was lower than that of the treatment group, and in the prevention group, the MDA content of the high concentration experimental group was comparable to that of the V_C_ group. This indicates that a certain concentration of fermentation liquid has the ability to prevent cellular oxidation in advance. SOD as a molecule of the antioxidant defense system in organisms (Ighodaro and Akinloye (2018), can scavenge oxygen free radicals to maintain the balance of oxidation and antioxidation in the body to evaluate the antioxidant capacity of fermentation liquid. In Figure 3G, the SOD activity in the experimental group was significantly increased compared to the model group (P < CAT, a typical reactive oxygen species (ROS) scavenging enzyme, can promote the decomposition of H_2_O_2_ to protect the body from ROS (Gayashani Sandamalika et al., 2021). In Figure 3H, there was a significant increase in CAT activity in the fermentation liquid and V_C_ groups compared to the model (*p* < 0.05) and the treatment group was more effective than the prevention group. As the concentration of fermentation liquid increased, the CAT activity increased, and at a fermentation liquid concentration of 2%, the CAT activity in the treatment and prevention groups was comparable to that in the V_C_ group.

### 3.3. Functional components in *Dendrobium* fermentation liquid

#### 3.3.1. Polysaccharides in *Dendrobium officinale*

**Figure 4:**
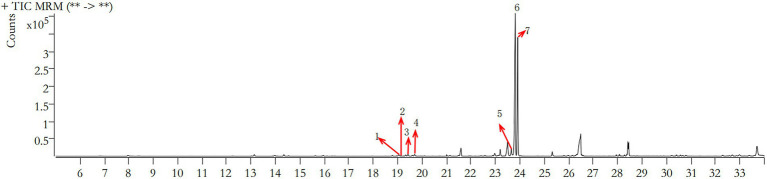
GC–MS total ion chromatogram of *Dendrobium* polysaccharides.

The retention time (RT) of polysaccharides in the fermentation liquid of *Dendrobium officinale* was concentrated in the range of 19–24 min by GC–MS, and the comparison with the metabolomics database revealed the presence of seven polysaccharides, namely rhamnose, ribose, arabinose, xylose, mannose, glucose, and galactose (Figure 4), among which glucose and galactose were the most abundant, at 194.628 and 103.899 μg/ml, respectively (Table 5). This indicates that polysaccharides, as one of the main active components contained in *Dendrobium* (Chen et al., 2021), mainly contained glucose and galactose in the fermentation liquid.

**Table 5:** Polysaccharide types and concentrations.

No.	Retention time t_R_ (min)	Peak height	Molecular formula	compound	Concentration/(μg/ml)
1	19.052	947.00	C_6_H_12_O_5_	Rhamnose	4.405
2	19.134	580.67	C_5_H_10_O_5_	Riboose	0.334
3	19.331	1191.65	C_5_H_10_O_5_	Arabinose	1.779
4	19.691	1779.09	C_5_H_10_O_5_	Xylose	3.619
5	23.647	9224.25	C_6_H_12_O_6_	Mannose	6.156
6	23.815	133409.00	C_6_H_12_O_6_	Glucose	194.628
7	23.914	133761.00	C_6_H_12_O_6_	Galactose	103.899

#### 3.3.2. Flavonoids in *Dendrobium officinale* fermentation liquid

**Figure 5:**
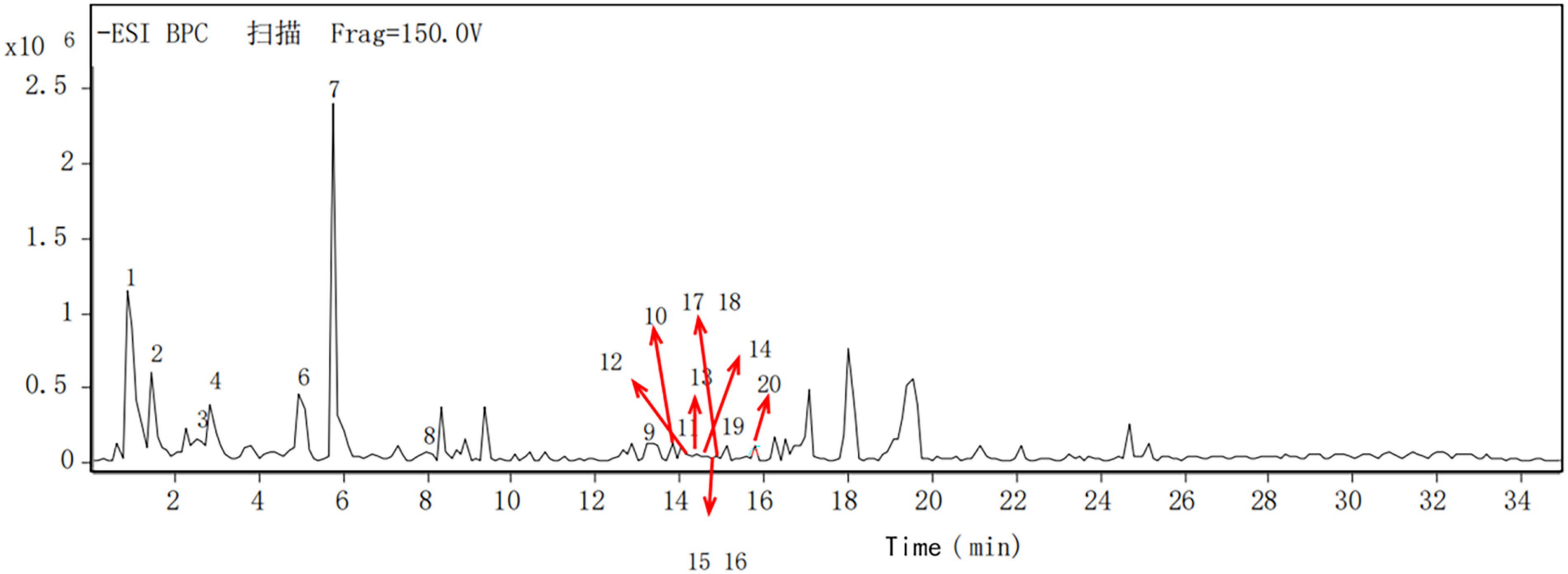
Total ion chromatogram of *Dendrobium* flavonoids extract at negative mode.

**Figure 6:**
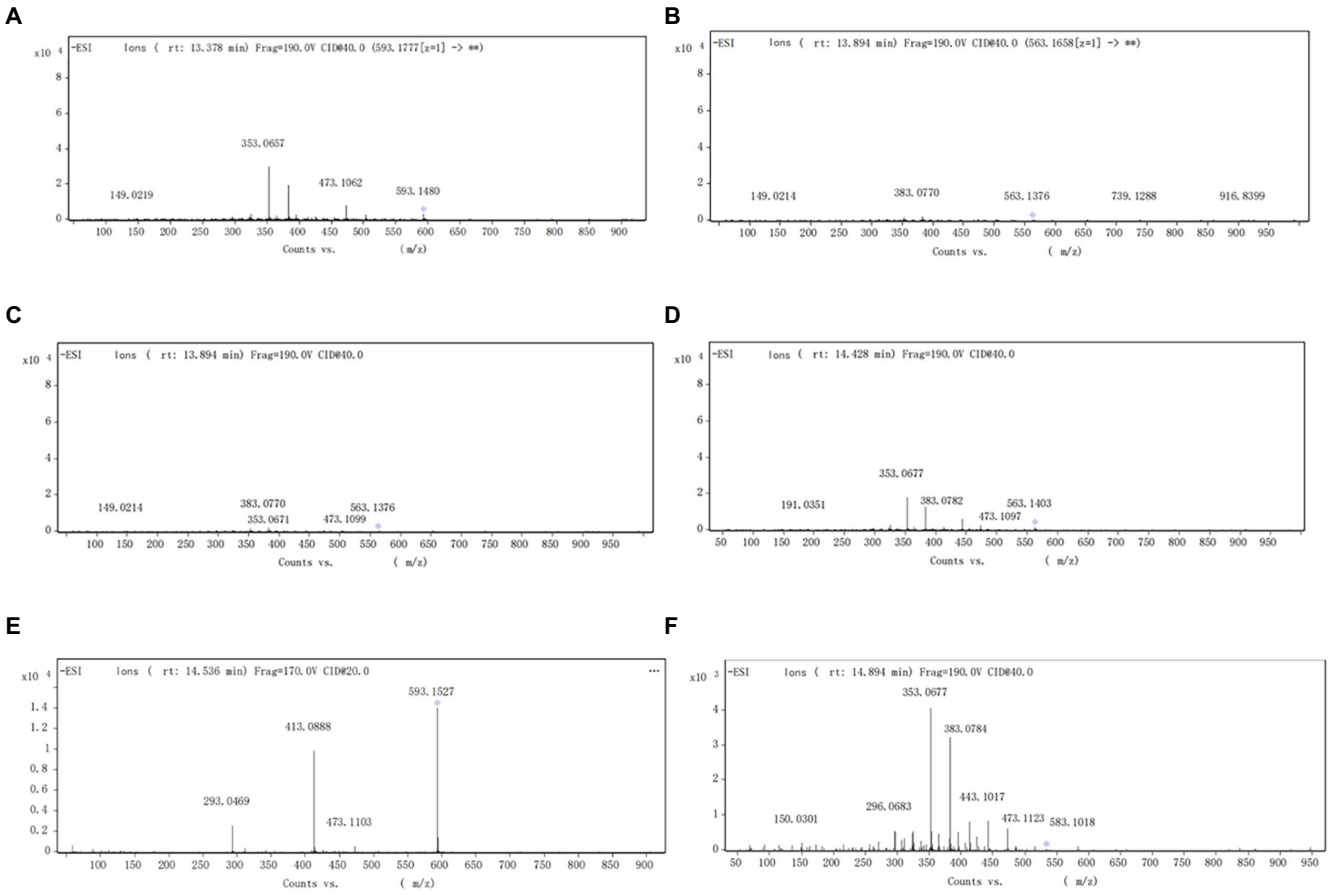
**(A)** Secondary mass spectrum of vitzenin-2 (apigenin-6,8-C-β-D-diglucoside). **(B)** Secondary mass spectrum of vitzenin-1 (apigenin-6-C-β-D-xylose-8-C-β-D-glucoside). **(C)** Secondary mass spectrum of schaffoside (apigenin-6-C-β-D-glucose-8-C-α-L-arabinoside). **(D)** Secondary mass spectrum of isoschaftoside (apigenin-6-C-α-L-arabinoside-8-C-β-D-glucoside). **(E)** Secondary mass spectrum of vitexin glucoside. **(F)** Secondary mass spectrum of apigenin- 6-C-α-L-arabinopyranoside-8-C-β-D-xylopyranoside.

The components of the flavonoid extracts were analyzed by HPLC-Q-TOF-MS (Yue et al., 2022). A total of 20 ion flow peaks were detected with retention times and mass spectral information (Figure 5). Six flavonoid components were identified analytically (Table 6). By secondary spectroscopy of these compounds, The flavonoids in *Dendrobium officinale* fermentation liquid appeared as the mass-to-charge ratio (m/z) of 473 [M-H-90]^−^, 383 [M-H-180]^−^, and 353 [M-H-180]^−^ of apigenin characteristic fragment ions (Figure 6). This indicates that the flavonoids in the fermentation liquid of *Dendrobium officinale* are mainly structured with apigenin glycoside elements.

**Table 6:** Flavonoids in *Dendrobium officinale* fermentation liquid.

No	Retention time t_R_ (min)	[M−H]^−^ (m/z)	Ion fragment (m/z)	Molecular formula	Compound
9	13.378	593.148	353, 383, 473, 503	C_27_H_30_O_15_	Vicenin-2
10	13.894	563.140	353, 383, 443, 473, 395,297, 325	C_26_H_28_O_14_	Vicenin-1
11	14.036	563.140	353, 383, 443, 473, 297, 325	C_26_H_28_O_14_	Schaffoside
14	14.428	563.140	353, 383, 443, 473	C_26_H_28_O_14_	Isoschaftoside
16	14.536	594.156	593, 473, 413, 293	C_27_H_30_O_15_	glucosylvitexin
18	14.894	533.131	473, 443, 383, 353	C_25_H_26_O_13_	Apigenin 6-C-α-L-arabinopyranosyl-8-C-β-D-xylopyranoside

#### 3.3.3. Polyphenols in *Dendrobium officinale* fermentation liquid

**Figure 7:**
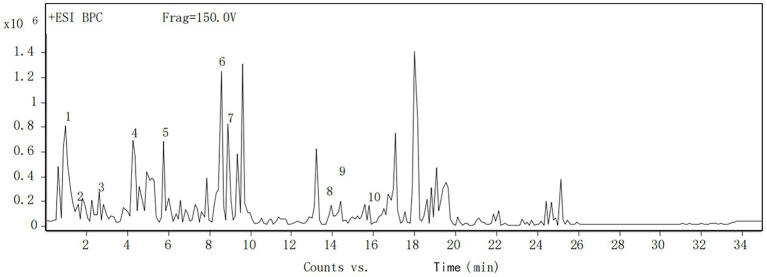
Total ion chromatogram of *Dendrobium* phenol extract at negative mode.

The fermentation liquid was analyzed by the HPLC-Q-TOF-MS technique (Li et al., 2017) and a total of 10 ion flow peaks were detected with retention time and mass spectrometry information (Figure 7). Four phenolic acid components were identified by comparing the metabolomics database, namely gallic acid, protocatechuic acid, catechol, and sessile pentosidine B (Table 7).

**Table 7:** Polyphenols in the fermentation liquid.

No	Retention time t_R_ (min)	[M−H]^−^ (m/z)	Ion fragment (m/z)	Molecular formula
5	5.011	169.015	C_7_H_6_O_5_	Gallic acid
6	8.400	153.019	C_7_H_6_O_4_	Protocatechuic acid
7	8.590	109.030	C_6_H_6_O_2_	Catechol
10	15.789	598.250	C_28_H_36_O_13_	Sessile glycoside B

## 4. Conclusion

Based on single-factor experiments, lactose content, fermentation temperature, and stock ratio were identified as the factors that significantly influenced the fermentation of *Dendrobium*. The optimal fermentation conditions for *Dendrobium* were obtained through orthogonal experiments: lactose content of 7.32%, fermentation temperature of 37.50°C, and a material-liquid ratio of 1:4. In this study, *in vitro* antioxidant tests were conducted on the fermentation liquid, and it was found that *Dendrobium* fermentation liquid had good antioxidant capacity, effectively reduced MDA content in human fibroblasts, and increased SOD and CAT activity, indicating that *Dendrobium* fermentation liquid had good therapeutic and preventive capacity. It was found that *Dendrobium* can effectively reduce MDA content and increase SOD and CAT activity in human fibroblasts. When the concentration of fermentation liquid is 2%, the antioxidant ability of fermentation liquid is close to that of V_C_. The components of the fermentation liquid were analyzed for polysaccharides, flavonoids, and phenolics. The polysaccharides were found to include rhamnose, ribose, arabinose, xylose, mannose, glucose, and galactose, with the highest glucose and galactose contents of 194.628 and 103.899 μg/ml, respectively. The flavonoids all contain characteristic fragment ions of apigenin with mass-to-charge ratios (m/z) of 473[M-H-90]^−^, 383[M-H-180]^−^, and 353[M-H-180]^−^, indicating that the fermentation liquid of *Dendrobium officinale* contains mainly flavonoids with apigenin glycosides as the basic structure. Finally, the fermentation liquid was also detected to contain four phenolic acid compounds based on gallic acid, protocatechuic acid, catechol, and sessile pentosidine B. The isolation and purification of the specific components of the fermentation liquid will be reported in the next study and their secondary and tertiary structures as well as other biological activities such as whitening, antibacterial, and antitumor will be confirmed in further studies.

## Data availability statement

The original contributions presented in the study are included in the article/supplementary material, further inquiries can be directed to the corresponding authors.

## Author contributions

All authors listed have made a substantial, direct, and intellectual contribution to the work and approved it for publication.

## Funding

This research has been funded by Natural Science Foundation of China [grant number 31400680], Science and Technology Plan Project of Guangzhou [grant number 201802030009]. This study was supported by National Key Clinical Specialty Construction Project (Clinical Pharmacy) and High level Clinical Key Specialty (Clinical Pharmacy) in Guangdong Province.

## Conflict of interest

SL, JY, WX, and YX were employed by Guangzhou Base Clean Cosmetics Manufacturer Co., Ltd., GY was employed by CAS Testing Technical Services (Guangzhou) Co., Ltd., YR was employed by Guangzhou Huashuo Biotechnology Co. Ltd.

The remaining authors declare that the research was conducted in the absence of any commercial or financial relationships that could be construed as a potential conflict of interest.

## Publisher’s note

All claims expressed in this article are solely those of the authors and do not necessarily represent those of their affiliated organizations, or those of the publisher, the editors and the reviewers. Any product that may be evaluated in this article, or claim that may be made by its manufacturer, is not guaranteed or endorsed by the publisher.
