# Corrigendum: Improvement of antioxidant activity and active ingredient of *Dendrobium officinale via* microbial fermentation

**DOI:** 10.3389/fmicb.2023.1179511

**Published:** 2023-03-16

**Authors:** Gen Yu, QingFen Xie, WenFeng Su, Shuang Dai, XinYi Deng, QuLiang Gu, Shan Liu, JeonYun Yun, WenHao Xiang, Yang Xiong, GuanDong Yang, Yifei Ren, He Li

**Affiliations:** ^1^Key Specialty of Clinical Pharmacy, The First Affiliated Hospital of Guangdong Pharmaceutical University, Guangzhou, China; ^2^Guangdong Provincial Key Laboratory of Pharmaceutical Bioactive Substances, School of Basic Courses, Guangdong Pharmaceutical University, Guangzhou, China; ^3^Guangzhou Base Clean Cosmetics Manufacturer Co., Ltd., Guangzhou, China; ^4^CAS Testing Technical Services (Guangzhou) Co., Ltd., Guangzhou, China; ^5^Guangzhou Huashuo Biotechnology Co., Ltd., Guangzhou, China

**Keywords:** *Dendrobium officinale*, fermentation, condition optimization, antioxidant, GC–MS

In the published article, there was an error in affiliations 5 and 6. Instead of “^5^School of Life Sciences and Biopharmaceuticals, The First Affiliated Hospital of Guangdong Pharmaceutical University, Guangzhou, China and ^6^Guangzhou Huashuo Biotechnology Co., Ltd., Guangzhou, China,” the 5th affiliation should be replaced by the 6th “^5^Guangzhou Huashuo Biotechnology Co., Ltd., Guangzhou, China.”

In the published article, there was an error regarding the affiliation for He Li. Instead of affiliation 5, they should be affiliated with 1, “Key Specialty of Clinical Pharmacy, The First Affiliated Hospital of Guangdong Pharmaceutical University, Guangzhou, China.”

The authors apologize for these errors and state that this does not change the scientific conclusions of the article in any way. The original article has been updated.

## Publisher's note

All claims expressed in this article are solely those of the authors and do not necessarily represent those of their affiliated organizations, or those of the publisher, the editors and the reviewers. Any product that may be evaluated in this article, or claim that may be made by its manufacturer, is not guaranteed or endorsed by the publisher.

